# Nitrite-Free Implications on Consumer Acceptance and the Behavior of Pathogens in Cured Pork Loins

**DOI:** 10.3390/foods11060796

**Published:** 2022-03-10

**Authors:** Luis Patarata, Filipa Carvalho, Maria João Fraqueza

**Affiliations:** 1 CECAV, Animal and Veterinary Research Center, Associate Laboratory for Animal and Veterinary Sciences (AL4AnimalS), University of Trás-os-Montes e Alto Douro, 5001-801 Vila Real, Portugal; fc7396975@gmail.com; 2 CIISA, Centre for Interdisciplinary Research in Animal Health, Faculty of Veterinary Medicine, Associate Laboratory for Animal and Veterinary Sciences (AL4AnimalS), University of Lisbon, Avenida da Universidade Técnica, 1300-477 Lisboa, Portugal; mjoaofraqueza@fmv.ulisboa.pt

**Keywords:** nitrite, sensory, color, *Clostridium*, *Listeria monocytogenes*, *Salmonella*, cured loin, cured meat product

## Abstract

Cured pork loins are valued products due to their particular sensory characteristics. These products are usually prepared with nitrite to guarantee adequate color and pathogen control. The use of nitrite in meat products has been criticized due to its potential contribution to carcinogenic N-nitroso-compound formation. The present work aimed to evaluate the effect of eliminating nitrite from the manufacturing of cured loins made with wine- and water-based marinades on the color evaluation of consumers and on the behavior of *Clostridium sporogenes*, *Listeria monocytogenes*, and *Salmonella*. The use of nitrite in processing cured loins resulted in a color considered adequate by more than 50% of the consumers. When nitrite was not used, the color was described mainly as weak. The hedonic evaluation of cured loins did not reflect the color evaluation. The samples with a weak and an adequate color had similar hedonic evaluations. The present work did not allow us to infer the potential interest in injecting *S. xylosus* into meat to prepare cured loins. The use of nitrite did not affect the survival of *Cl. sporogenes*, *L. monocytogenes*, or *Salmonella*. The reduction in the aw was the primary determinant influencing pathogen survival. The production of nitrite-free cured loins seems possible once the control of pathogens can be achieved. However, the product will have a weaker color. Consumers appreciate sensory aspects other than color, which, combined with the positive impact of the “additive-free” claim, can support the possibility of producing cured loins without nitrite.

## 1. Introduction

The use of nitrite as a food additive in meat products has been a common practice since the middle of the last century. Its use is justified mainly by its, until now, irreplaceable action on stabilizing the typical color of cured meat products and controlling biological hazards, particularly *Clostridium botulinum*. It is also an antioxidant and is involved in flavor formation [1]. The regulations on its use have evolved according to scientific knowledge on its benefits and potential health risks. In 2015, the International Agency for Research on Cancer (IARC) disclosed a report revealing the trends found in a meta-analysis of the relationship between the consumption of processed meats and the occurrence of colon cancer [2]. That association was found to be clearly demonstrated—that is why processed meats have been classified as Group I risk factors, with a mean odds ratio of 1.18, indicating an increase of 18% in the probability of having colon cancer for processed-meat consumers, when compared with the risk of non-consumers [3]. The N-nitro compounds formed after the reaction of nitrite with amines that eventually become present in the product and the presence of polycyclic aromatic hydrocarbons (PAHs) in smoked products are the main hazards linked to the carcinogenic risk of processed meats [4]. Considering the established relationship and the relative risk—which is not exceptionally high—there has been a trend to reduce nitrite in meat products [5,6,7]. This trend is warrantable, since nitrite-cured meat products might be unsafe for consumers’ health due to the presence of potential chemical hazards. However, the opposite is true: the absence of nitrite might also be potentially harmful for different reasons, mainly due to the potential presence of biological hazards, namely *Cl. botulinum* and *L. monocytogenes* [8,9].

When reducing or eliminating nitrite from meat products during manufacture, alternative measures should be considered to guarantee the strategy’s efficacy. The effect of nitrite on meat products’ sensory attributes is mainly related to the stabilization of color due to the formation of nitrosylmyoglobin by the bond of nitric oxide with the iron of that hemoprotein. The characteristic color of this pigment is stable during the manufacture and shelf life of the product [10]. Nitrite also contributes to the aroma by its antioxidant action and participation in forming several aromatic compounds [11,12]. The phenomena underlying the curing of nitrite-free meat products have been researched in the last years, and several clues are indicating the importance of the formation of zinc-protoporphyrin in long-cured products, such as cured hams or cured loins, resulting in products with a color similar to that obtained by using nitrite [13,14,15]. Another strategy to obtain cured meat products with an adequate color is to favor the microbial production of nitric oxide by bacteria equipped with NO synthase (NOS), such as several coagulase-negative *Staphylococcus* (CNS) strains used in meat products. In conventional use, nitrite is reduced to NO, the chemical molecule of interest for the formation of nitrosylmyoglobin and one of the more important for inhibiting pathogens. When CNS with NOS activity is involved, the NO produced can bind the myoglobin to form the cured pigment without adding nitrite to the product [16,17]. The aroma of cured meat products is due to a certain level of lipid oxidation and the microbial catabolism of amino acids [18,19,20]. The aroma is also highly determined by the seasonings used, particularly by smoke, if this operation is used [21,22].

From the safety point of view, it is necessary to consider the time necessary to achieve a safe water activity (aw) that does not allow pathogen growth or toxinogenesis. The minimum aw needed to inhibit the *Cl. botulinum* toxinogenesis is around 0.95 [23,24]. In cured sausages, particularly those with a smaller caliber, it can be achieved in the first days of drying [25,26]. The inhibition of other pathogens, namely the Gram-negative bacteria, might also be achieved at these levels of drying [6,27,28], and the main concern is mainly associated with the potential growth of *Listeria monocytogenes* [27,29,30] and the eventual production of enterotoxins by *Staphylococcus aureus* [31].

Cured loins are meat products included in the vast tradition of dry-cured meat products made in the Mediterranean region and elsewhere. The recipes vary between regions in line with the local gastronomic traditions [5,32,33,34,35]. It is common, in wine-producing regions, to season the meat with wine, both for cooking and dry-cured sausage production [36,37]. In the Portuguese Douro region, with a long wine-making tradition, the preparation of cured loins includes cutting the pork loins transversally to the muscles’ orientation (300–500 g each piece), then seasoning them in a marinade composed of red wine, pure or diluted with water, salt, garlic, and bay leaves. The meat is maintained in the marinade for 2 to 7 days at low temperatures. The meat is filled into pork gut, tied, smoked, and dried for 2 to 4 weeks until it reaches a safe aw [38]. When their industrial production includes nitrite, the meat is salted with a mixture of salt and nitrite for 24 h before the marinade. The practice of marinating in wine to produce dry-cured sausages results in a characteristic winy flavor and a red-burgundy color when the wine is red [39,40]. Additionally, due to its composition of ethanol, organic acids, and phenolic compounds, it is expected that the wine marination procedure will reduce microbial growth on the meat [41,42,43,44]. Whole pieces of meat are usually used, and they can be marinated at low temperatures for several days. This acidic environment might also regulate the microbiota in the cured loins, as it favors the competition of the acidophilic lactic acid bacteria (BAL) while inhibiting several pathogens that may be present [45,46].

The present work aimed to evaluate the effect of eliminating nitrite from the manufacturing of cured loins made with wine-based and water-based marinades on the color and the behavior of *Clostridium sporogenes*, *Listeria monocytogenes*, and *Salmonella*.

## 2. Materials and Methods

### 2.1. Experimental Design

The experiment comprised two independent parts. In the first part, we prepared cured loins for sensory evaluation. In the second, we prepared a challenge test to independently evaluate the three pathogens’ behavior. Each part of the experiment included three batches prepared with meat from three different suppliers.

### 2.2. Microorganisms and Culture Conditions

The *Staphylococcus xylosus* used to test its potential to redden the cured loins was previously isolated and identified from the natural microbiota of nitrite-free cured loins made by small producers in a mountain region [47]. For the microbial challenge test, we used *Cl. sporogenes* DSM 767, *L. monocytogenes* ATCC 35152, and *Salmonella* ATCC 49214. *Cl. sporogenes* was used as a surrogate for *Cl. botulinum* [48]. For *L. monocytogenes* and *Salmonella*, a mixture was prepared of the collected strain and two wild strains isolated from meat products. Pure cultures of the *L. monocytogenes* or *Salmonella* strains were combined for the inoculation. The cultures were prepared according to the process described in the reference [37].

### 2.3. Sensory Analysis Experiment

#### 2.3.1. Preparation of Cured Loins for Sensory Evaluation

Pork loins were cut transversely into portions of around 350 g each. The meat was divided into three groups: (1) salted with 2% salt, (2) salted with 2% salt and 150 mg/kg of sodium nitrite (included at 5% in salt, Formulab, Portugal), and (3) previously injected with 20 mL of a suspension of an indigenous *S. xylosus* (around 6 log CFU/mL) and salted with 2% of salt. The meat without *S. xylosus* was injected with 20 mL of water. The meat rested at 4 °C for 24 h. Each group was further divided into two: one portion to be marinated in a mixture of 50% red wine from the region, 50% water, 1% fresh chopped garlic, and 0.5% dried bay leaf; in the other part, the wine was replaced by water (Table 1). The ratio of meat to the marinade mixture was approximately 1:1 (*w*/*v*).

The loin pieces were filled into collagen casing (Fibraco, Poland) and smoked (<35 °C) in a chamber with an electric smoke generator using beechwood chips for 8 h. The drying phase was performed over 30 days in a climatic chamber at 15 ± 2 °C with an initial relative humidity (RH) of 95% and consecutive reduction to maintain the RH at 5% below the aw. Samples were then vacuum-packaged until the sensory analysis, which occurred within 2 weeks.

#### 2.3.2. Sensory Analysis

In-person test with cured loin samples. A consumer test was performed with 104 volunteers recruited from the personal and professional contacts of the authors. The test included a hedonic evaluation, yes/no purchase intentions, and a Just About Right (JAR) scale. The hedonic evaluation was conducted on a 9-point scale (1—strongly dislike, 9—strongly appreciate) to answer the question, “How would you classify the cured loin we tested?” The 5-point JAR scale was used to evaluate the adequacy of the color, ranging across much too weak (−2), weak (−1), ideal (0), strong (+1), and much too strong (+2). The evaluations below and above the ideal were condensed into a single class—weak or strong [49]. The samples from the three batches were combined, and each consumer only tested one sample from each treatment/batch. Each batch was randomly distributed across the consumers (a total of 6 samples per consumer). The consumers were 66% women, aged between 19 and 66 years old, and were usually consumers of cured meat products (81%).

**Figure 1:**
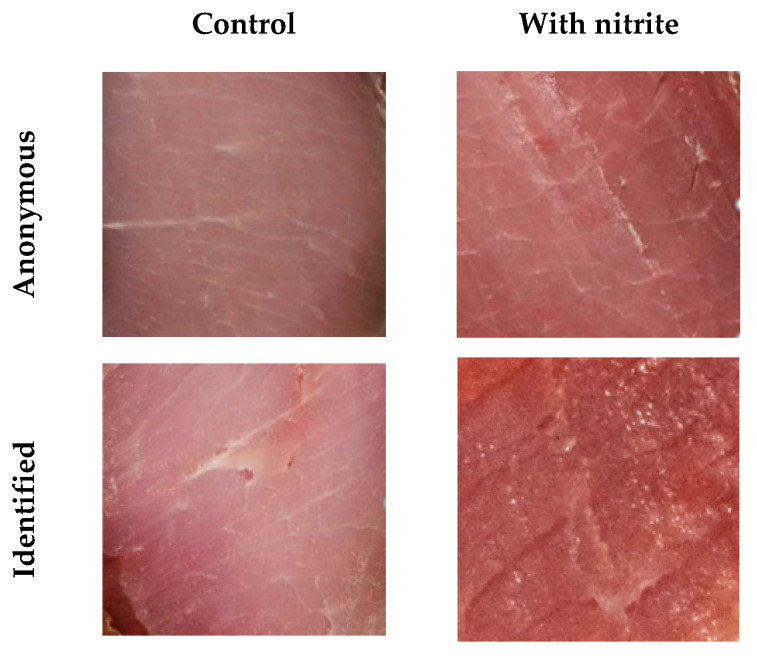
Images of cured loins presented in the online consumer test. Anonymous—no information was provided to the consumer; identified—samples were identified with the information “Cured Loin 1 has a food additive indicated by the WHO as being carcinogenic; Cured Loin 2 does not have any chemical additives”.

Online test with cured loin images. This test was performed to evaluate the effect of information on the presence of nitrite on the preference for cured loins based on color adequacy. This test was performed only with samples from two experimental conditions, with or without nitrite, made with wine-based marinades. Cured loins were cut transversally and photographed (Figure 1) in standardized condition, as defined in [50].

An online test was made using the web platform GoogleForms. In the first part, two images were presented to the consumer, who asked, “Which of the cured loins has a more characteristic color?” In the second part, the samples were identified with the phrase “Cured Loin 1 has a food additive indicated by the WHO as being carcinogenic; Cured Loin 2 does not have any chemical additives”, and it was asked again which of the two products had a more characteristic color. The respondents (*n* = 168) of the online test were 74% women, aged between 18 and 62 years old, and were usually consumers of cured meat products (89%).

#### 2.3.3. Instrumental Evaluation of Color

Due to the theoretical instability of the wine pigments, particularly in the presence of nitrite, the instrumental color was measured in cured loins at 8 and 30 days of drying to determine the color’s stability. The L*a*b* color parameters were measured on the surface of the cured loins’ longitudinal section with a Minolta CR 310 chromameter with a standard D65 illuminant. The measuring head of the chromameter (a 50 mm diameter) used a wide area of illumination and a 0° viewing angle. Measurements were taken in triplicate immediately after cutting the samples across the central part of the sample.

### 2.4. Microbial Challenge Test

#### 2.4.1. Preparation of Cured Loins for the Microbial Challenge Test

For this part of the experiment, the formulations tested were the same as indicated in Table 1, only with some modifications regarding the previous preparation of the meat, as formerly described [33]. After cutting the meat in a safety cabinet with sterile tools, the pieces were exposed to UV light for 15 min on each side and frozen until use. The meat portions were thawed for 48 h at 4 °C. The meat was inoculated with the pathogens in a laminar flow chamber. Each microorganism culture was diluted in 1 L of 0.85% NaCl to obtain a concentration of about 6 log bacteria/mL. The contamination was carried out in three different blocks with the respective microorganisms: *Cl. sporogenes*, *L. monocytogenes*, or *Salmonella*. The meat portions were dipped into the suspension of each microorganism for about 1 min and shaken manually. The pieces of meat were then removed from the suspension and placed on steel net trays with a ~2 cm mesh to drip off the excess suspension. The meat, in polyethylene bags, rested at 4 °C for 18 h and was then rinsed in 0.85% NaCl to withdraw the microorganisms that had not adhered. Preparation of the cured loins was as indicated in Section 2.3.1. Samples were taken for analysis at the filling phase and at 8, 21, and 30 days after smoking. One cured loin was used for each sampling time in each batch for each challenging microorganism.

#### 2.4.2. Activity of Water and pH

Water activity was measured in a Rotronic Hygroscope DT WA-40 (Bassersdorf, Switzerland). The pH was measured directly in the cured loins with a penetration electrode using a pH meter (model MicropH 2002, Crison, Barcelona, Spain).

#### 2.4.3. Microbial Analysis

At the defined sampling times, one cured loin was collected. The casing of the cured loin was aseptically removed, and 10 g of the sample was excised from the outer part of the piece (~0.5 cm from the surface) in different locations of the piece. An initial dilution of 1:10 was prepared in a 0.85% NaCl solution, followed by homogenization for 3 min in a stomacher. When low counts were expected, to increase the detection limit, the initial dilution was prepared at 1:5. Counts of *Cl. sporogenes* were made after pasteurization (75 °C, 20 min) of the initial dilution. The count was made by incorporating 1 mL in PA3679 medium [51] after 4 days of incubation at 30 °C. *L. monocytogenes* and *Salmonella* were counted in CHROMagar media (Paris, France) by spreading 0.1 mL on the surface. When low counts were expected, 0.5 mL was spread in two Petri dishes (0.25 mL each). The first was incubated at 30 °C for 48 h and the second was incubated at 37 °C for 24 h. In the absence of *Salmonella* growth after 24 h, the dishes were incubated for a further 24 h. Counts of CNS were made in Mannitol Salt Agar (Biokar, France) at 30 °C for 48 h. The results are presented as log CFU/g.

### 2.5. Data Analysis

The results from the continuous variables—hedonic evaluation and the number of surviving microbes in the microbial challenge test—were compared by ANOVA. The Chi-square test compared the results of consumption intention. The penalty analysis was calculated to evaluate the impact of an evaluation of non-ideal color on the hedonic evaluation. The changes in the evaluation of characteristic color between the anonymous and the identified presentation were tested by the McNemar test. The significance level used was 0.05. Data analysis was conducted using XLStat software (Addinsoft, Paris, France).

## 3. Results and Discussion

The cured loins presented a mean pH of 5.60 ± 0.20 and an aw of 0.87 ± 0.01. No significant differences (*p* ≥ 0.05) were observed between the six tested formulations. In non-inoculated cured loins, CNS counts were low: 1.65 ± 1.30 log CFU/g at the filling stage, 0.80 ± 1.20 log CFU/g and 0.82 ± 1.23 log CFU/g after 8 and 21 days of drying, respectively; at 30 days of drying, these bacteria were not detected in non-inoculated samples. In inoculated samples, the counts were 3.93 ± 0.22 log CFU/g at filling and remained between 3.5 and 4.0 log CFU/g until the end of processing. No differences (*p* = 0.33) were detected between inoculated cured loins marinated in wine-based and water-based marinades (data not shown in the tables or figures).

### 3.1. Sensory Evaluation

The results of the hedonic evaluation are presented in Table 2. All the samples were classified above the center of the scale (5 in the 9-point scale). The general evaluation between the different formulations was close, with statistical differences found only between the cured loins marinated with water with nitrite, which had the highest score, and the control marinated in water, which had the the lowest. The hedonic evaluations of any other comparisons were similar. The consumption intention followed, as foreseen, the same trend as hedonic evaluation (Table 3).

**Figure 2:**
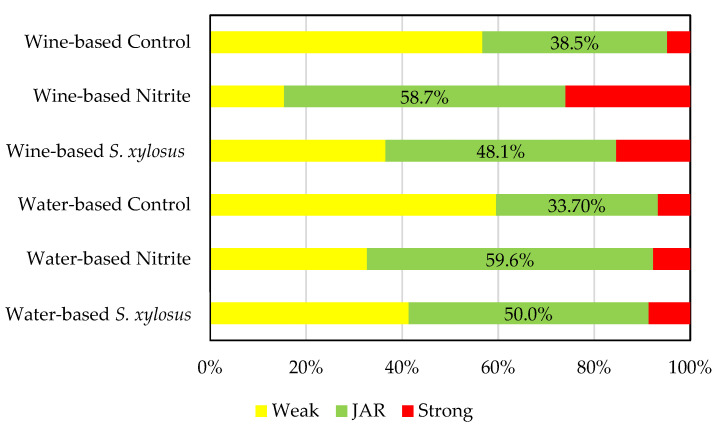
Just About Right evaluation of cured loins.

The JAR results are presented in Figure 2. In the two formulations including nitrite, the color was evaluated as ideal by more than 50% of consumers. The cured loins made with *S. xylosus* in a water-based marinade were evaluated as having an ideal color by 50% of the consumers. The limit of 50% of consumers evaluating the characteristic as about right is commonly used to determine if a product is ready to be launched in the market, considering that weak or strong characteristics prejudice the hedonic evaluation [52].

As shown in Table 4, the relationship between the JAR evaluation and the hedonic evaluation was established through penalty analysis.

In cured loins made with nitrite and the water-based marinade, there was a penalty associated with weak color of 0.77 units in the nine-point hedonic evaluation. The cured loins made with nitrite and the wine-based marinade had a negative penalty (−0.40) that represented a gain. On average, 26.0% of consumers who evaluated the color as strong gave a hedonic evaluation higher than that found for those considering the color to be about right. The JAR evaluation of color was at or below 50% for all the other formulations. In all of these cases, weak color was the cause of lowering the hedonic evaluation by 0.91 to 1.32 units.

Some results might seem incoherent. For example, for cured loins made with wine-based marinades without nitrite, 56.7% of the consumers considered these products to have a weak color (Figure 2), but the mean hedonic evaluation and the consumption intentions were high. Cured loins made with nitrite and wine-based marinades, despite having a high percentage of consumers (58.9%, Figure 2) considering the color to be adequate, had a low hedonic evaluation and the lowest consumption intentions (53.8%). It is necessary to consider that the penalty analysis compares the mean hedonic scores for the samples classified as having a JAR color and the hedonic scores for the weak and strong groups. It is also necessary to consider that the hedonic evaluation was an open question, allowing the consumer to perform a multifactorial evaluation and not focus only on the color. Ultimately, odor or flavor aspects could have driven the consumers’ evaluation more than the color.

Consumers might be influenced by a perception of the “naturalness” of the food [53]. We asked 164 consumers to indicate, using an online test, between two cured loin images, which one they considered to have a more characteristic color. In the first instance, we presented the images anonymously; in the second moment, they were identified as additive-free or having a carcinogenic chemical additive (Table 5).

A general trend was observed of considering the images of cured loins without nitrite to have a more characteristic color when the cured loins were anonymous (58.5%). This evaluation increased (*p* < 0.05) to 67.1% when the images were identified. Almost 13% of the consumers changed their evaluation of “more characteristic color” for the nitrite-free cured loin images. The trend observed in this test should not be compared with the results from the hedonic JAR evaluation, as the natural variability among samples and slices in tests made with live consumers did not exist in this online test with images, where the image was always the same. Nonetheless, a potential suggestion effect was observed. Consumers’ choices and general hedonic evaluation depend highly on personal credence cues [54]. Regarding the clean-label issue [55], there have been several studies on trendy aspects of food quality, such as organic and animal welfare [56,57], that have tended to find similar results, which were highly dependent on personal expectations, as the perception is the result of a sensory stimulus and the related cognitive processing [58,59].

### 3.2. Instrumental Evaluation of Color

The instrumental color of cured loins (Table 6) was influenced by several factors, relating mainly to the nitrite and the stability of wine pigments, and probably by the interaction between both.

The color parameters presented in Table 6 show considerable differences between the tested formulations, after both 8 and 30 d of drying, with different trends in the first and second analysis period. After 8 d of drying, the cured loins made with nitrite had a redder color than any other formulation, both in wine and water-based marinades. These samples also had a more yellow color. Nitrosylmyoglobin has a clear red color, reflected in the a* values [60]. After 30 d of drying, the a* values from cured loins prepared with nitrite in a water-based marinade showed the reddest color (13.05 ± 1.09), but those prepared with wine and nitrite had a lower a* (8.60 ± 0.41). This reduction in a* might be associated with meat pigment modification due to the presence of wine or to the masking effect of wine pigments. In samples with a lower a*, the b* parameter was higher.

Considering the control cured loins and those prepared with nitrite, this suggests that the yellow component of color was driven by the presence of wine, probably derived from the wine pigments [61]. Anthocyanins are the major wine compounds contributing to its characteristic red-purple color, with a minor contribution of tannins [62]. The stability of anthocyanins is highly dependent on pH and the reaction with other phenolic compounds or oxygen, among other factors. When red wine is used to marinate meat, its color might change due to changes in the anthocyanin oxidation status or a reaction with other compounds [61]. The higher pH found in meat and also in the cured loins, compared with the pH of wine, could result in its chemical modification, leading to browner compounds. How nitrite can interact with the wine color is not clear. Anthocyanins are recognized as being able to neutralize reactive radical species by electron or hydrogen atom transference from phenolic groups, resulting in color modification [63,64]. The various reactive species formed during the nitrite reduction, namely peroxynitrite (ONOO−), might also be involved in this complex interaction between nitrite and anthocyanins in cured loins [23,65], resulting in the oxidation of anthocyanins and the associated color change. We believe that these reactions associated with color modification would eventually slow, as the results after 8 d of drying did not suggest these modifications. The use of *S. xylosus* resulted in slight differences in the color parameters. The a* was similar to that of the control, and the b* had only slight differences. The potential production of nitric oxide by CNS has been tested in culture media and in minced meat [66,67]. The possibility of having microbially produced nitric oxide in a single-piece product such as cured loins does not seem to be effective, mainly because the bacteria fail to grow to high counts, which could reveal its potential.

### 3.3. Microbial Challenge Tests

The challenge test results for *Cl. sporogenes*, *L. monocytogenes*, and *Salmonella* are presented in Table 7. It was observed that during the 5 days of marination, *Clostridium* remained at a level similar to that observed after inoculation in the control samples. No effect of using wine in the marinade was observed. Both the presence of nitrite and the competitive *S. xylosus* resulted in a reduction (*p* < 0.05) in the microorganism count. The product was smoked and dried for 8 days, which reduced the counts by around two logarithmic units under all the experimental conditions. After 21 days of drying, it was not detected.

The experiments with *L. monocytogenes* began with a level of contamination of 4.43 ± 0.13 log CFU/g. At the end of 5 days of marination, the counts were nearly 1 log CFU/g lower than after inoculation. The effects of nitrite and wine were not observed at this phase. Only a slight difference (*p* < 0.05) was detected between the sample with the highest count (the control in the water-based marinade) and the sample inoculated with *S. xylosus* in the same marinade. Although *L. monocytogenes* is a psychotropic microorganism [68], it did not multiply during the marination phase, even when the conditions were favorable, such as a lack of nitrite or inoculated competition. This might be due to the initial high salt concentration at the surface until it equilibrated in the product, or a washing effect of the microorganisms might have occurred in the marinade, ultimately due to the ability of these strains, under these conditions, to adhere to the food surface [69]. The effect of smoking and drying on the pathogens’ survival was observed as a reduction in the counts of one to two logarithmic units after 8 days of smoking. In this phase, none of the effects studied was found to affect the survival of *L. monocytogenes*. The bacteria did not survive during the remaining drying period, as observed by the null counts after 21 days. A residual number of survivals was observed only in one experimental condition: the water-based marinade with *S. xylosus*.

The trend of *Salmonella*’s behavior was similar to that observed for *L. monocytogenes*, with no observable effects of the experimental conditions, but it was strongly influenced by smoking and drying. The counts remained similar to that in the inoculated meat after the marination, confirming the strong attachment ability of *Salmonella* to meat [70]. After 8 days of drying, the reduction in the counts of *Salmonella* was two to three logarithmic units. The resilience of *Salmonella* is patent in its presence after 21 days of drying under certain experimental conditions, but after 30 days of drying, it was not detectable. After 30 days of drying, no counts of any of the challenged microorganisms were observed in any of the experiments.

Cured loins are products made from a whole piece of meat. Once contamination occurs on the pieces’ surface, the preservation barriers are expected to have higher importance on the surface, contrary to cured products made from minced meat [71]. The contact with higher salt content at the beginning of the process, when it had not equilibrated in all the pieces [72], the eventual washing effect produced by the marinade, and the smoke compounds [73] contributed to the low survival of the inoculated microorganisms challenged in the present study. The reduction in aw, which was faster on the surface, also contributed to the lethal effect observed in the present work [25,74].

## 4. Conclusions

In processing cured loins, nitrite resulted in a color considered to be adequate by more than 50% of the consumers. The use of *S. xylosus* resulted in a 50% and 48.1% of consumers judging the color to be adequate when water-based or wine-based marinades were used, respectively. In the control samples, the color was considered adequate by less than 40% of the consumers. The hedonic evaluation of cured loins did not reflect the color evaluation, as samples considered to have a weak and an adequate color had similar hedonic evaluations. The results of the present work did not allow us to infer the potential interest in injecting *S. xylosus* in meat to prepare cured loins. Still, the JAR evaluation had a slightly higher proportion of consumers indicating that its color was adequate. The effect of being labeled as green or additive-free impacted consumers’ perceptions of the cured loins’ color. If consumers were informed about the presence of a potentially harmful additive, they tended to change their evaluation of characteristic color in additive-free products. From the results obtained in the present work, it is possible to infer that nitrite does not affect the survival of *Cl. sporogenes* (which was used as a surrogate for *Cl. botulinum*), *L. monocytogenes*, and *Salmonella*. The reduction in the aw was the primary determinant of pathogen survival.

The preparation of cured loins without nitrite should consider that the red color will be weaker, and when nitrite and wine are used simultaneously, color modifications can occur. Besides the color, consumers appreciated other sensory aspects. The multifactorial hedonic evaluation, combined with the positive impact of the “additive-free” claim can support the possibility of producing cured loins without nitrite.

## Figures and Tables

**Table 1:** Formulations tested in the experiment.

Marinades	(1) Control	(2) Nitrite	(3) *S. xylosus*
Wine-based (50 wine:50 water) 1% fresh garlic 0.5% bay leaves	2% salt	2% salt 150 mg/kg nitrite	2% salt 20 mL cell suspension
Water-based (100 water) 1% fresh garlic 0.5% bay leaves	2% salt	2% salt 150 mg/kg nitrite	2% salt 20 mL cell suspension

**Table 2:** Hedonic evaluation of cured loins. Results are presented as mean ± standard deviation.

Marinades	Control	Nitrite	*S. xylosus*
Wine-based	6.27 ± 1.38 ^ab^	5.81 ± 1.63 ^ab^	5.93 ± 1.66 ^ab^
Water-based	5.74 ± 1.63 ^b^	6.39 ± 1.42 ^a^	6.04 ± 1.71 ^ab^

^a, b^ Means followed by different letters are different (*p* < 0.05).

**Table 3:** Consumption intention of cured loins. Results are presented as a percentage of consumers and the standardized residuals.

Marinades	Control	Nitrite	*S. xylosus*
Wine-based	67.3 (0.82) ^1^	53.8 (−2.31)	68.3 (1.04)
Water-based	49.0 (−3.43)	76.0 (2.83)	68.3 (1.04)

^1^ Values in brackets are the standardized residuals.

**Table 4:** Mean drop (MD) and penalties associated with a non-ideal evaluation of the cured loins’ color.

Marinades		Control			Nitrite			*S. xylosus*		
	Level	%	MD	Penalties	%	MD	Penalties	%	MD	Penalties
Wine-based	Weak	56.7	**0.94 ^1^**	0.86	15.4	0.54	−0.050	36.5	**0.91**	0.71
	Strong	4.8	0		26.0	**−0.40**		15.4	0.24	
Water-based	Weak	59.6	**1.32**	1.34	32.7	**0.77**	0.78	41.4	**1.22**	1.35
	Strong	6.7	1.49		7.7	0.84		8.7	1.93	

^1^ Values marked in **bold** correspond to a mean drop obtained for the evaluation by at least 20% of consumers.

**Table 5:** Percentage of consumers considering the cured loins’ color to be more characteristic based on an observation of pairs ^1^ of online images in an anonymous test and after identification as having a carcinogenic additive or not (*n* = 164 consumers).

Presentation	Cured Loin Images	
	Without Nitrite	with Nitrite
Anonymous	58.5	41.5
Identified	67.1	32.9
Change between Anonymous and Identified		
Maintained	82.9	
Without → with nitrite	4.3	
With → without nitrite	12.8	
	*z* = 2.464, *p* = 0.013	

^1^ One image was from a cured loin with and the other without nitrite, but both with a wine-based marinade.

**Table 6:** Color parameters (L*a*b*) of cured loins after 8 and 30 d of drying. Results are presented as the mean ± standard deviation.

Parameter Marinades	Control	Nitrite	*S. xylosus*	Control	Nitrite	*S. xylosus*
		8 d Drying			30 d Drying	
L*						
Wine-based	58.41 ± 1.59 ^bc^	55.54 ± 2.19 ^c^	63.36 ± 1.28 ^a^	50.96 ± 0.38 ^a^	45.80 ± 1.09 ^b^	50.95 ± 0.42 ^a^
Water-based	59.07 ± 1.06 ^abc^	58.47 ± 1.83 ^bc^	60.28 ± 0.15 ^ab^	42.67 ± 1.35 ^c^	46.32 ± 1.99 ^b^	42.69 ± 0.42 ^c^
a*						
Wine-based	13.90 ± 1.34 ^b^	17.35 ± 0.48 ^a^	10.82 ± 0.85 ^c^	8.11 ± 0.57 ^c^	8.60 ± 0.41 ^c^	9.45 ± 0.17 ^bc^
Water-based	9.55 ± 0.92 ^c^	16.77 ± 0.74 ^a^	11.14 ± 0.13 ^c^	11.72 ± 1.13 ^ab^	13.05 ± 1.09 ^a^	9.32 ± 1.08 ^c^
b*						
Wine-based	5.14 ± 0.59 ^d^	8.01 ± 0.49 ^a^	5.64 ± 0.21 ^cd^	10.94 ± 0.08 ^ab^	10.50 ± 0.01 ^ab^	8.02 ± 0.50 ^c^
Water-based	6.53 ± 0.60 ^bc^	8.16 ± 0.50 ^a^	6.98 ± 0.22 ^ab^	7.60 ± 0.84 ^c^	9.37 ± 0.92 ^bc^	11.73 ± 1.36 ^a^

^a, b, c, d^ For each drying period, means followed by different letters are different (*p* < 0.05).

**Table 7:** Counts of *Cl. Sporogenes, L. monocytogenes*, and *Salmonella* in the challenge test made with cured loins. Results are presented as the mean and standard deviation.

		Marinades	Control	Nitrite	*S. xylosus*
*Cl. sporogenes*	Meat after inoculation		2.73 ± 0.23		
	Filling (5 d in the marinade)	Wine-based	2.60 ± 0.31 ^a^	1.84 ± 0.13 ^b^	1.83 ± 0.09 ^b^
		Water-based	2.97 ± 0.18 ^a^	1.80 ± 0.04 ^b^	1.64 ± 0.19 ^b^
	8 d after smoking	Wine-based	0.07 ± 0.12	0.08 ± 0.14	0.03 ± 0.06
		Water-based	ND	ND	0.01 ± 0.02
	21 d after smoking	Wine-based	ND	ND	ND
		Water-based	ND	ND	ND
					
*L. monocytogenes*	Meat after inoculation		4.43 ± 0.13		
	Filling (5 d in the marinade)	Wine-based	3.49 ± 0.14 ^b^	3.89 ± 0.03 ^ab^	3.81 ± 0.17 ^ab^
		Water-based	3.94 ± 0.06 ^a^	3.69 ± 0.01 ^ab^	3.50 ± 0.27 ^b^
	8 d after smoking	Wine-based	2.18 ± 0.58	2.75 ± 1.41	1.98 ± 0.28
		Water-based	2.06 ± 0.44	3.06 ± 1.15	1.55 ± 1.37
	21 d after smoking	Wine-based	ND	ND	ND
		Water-based	ND	ND	0.43 ± 0.74
					
*Salmonella*	Meat after inoculation		4.39 ± 0.03		
	Filling (5 d in the marinade)	Wine-based	4.41 ± 0.30	3.97 ± 0.36	4.24 ± 0.07
		Water-based	4.52 ± 0.14	4.05 ± 0.12	4.20 ± 0.16
	8 d after smoking	Wine-based	0.89 ± 0.78	1.21 ± 1.22	2.47 ± 0.06
		Water-based	2.75 ± 0.81	2.38 ± 0.30	2.56 ± 0.23
	21 d after smoking	Wine-based	1.37 ± 1.19	ND	ND
		Water-based	1.18 ± 0.31	0.48 ± 0.83	0.43 ± 0.37

^a, b^ Means followed by different letters are different (*p* < 0.05); ND—not detected.
